# Poly[[diaqua­tetra­kis­(μ_2_-benzene-1,4-di­carbonitrile-κ^2^ *N*:*N*′)iron(II)] bis­[tetra­chlorido­ferrate(III)] nitro­methane tetra­solvate]

**DOI:** 10.1107/S1600536812002486

**Published:** 2012-01-31

**Authors:** Pimonrat Prommon, Pinyada Promseenong, Kittipong Chainok

**Affiliations:** a Department of Chemistry, Faculty of Science, Naresuan University, Muang Phitsanulok 65000, Thailand

## Abstract

In the title compound, {[Fe^II^(C_8_H_4_N_2_)_2_(H_2_O)_2_][Fe^III^Cl_4_]_2_·4CH_3_NO_2_}_*n*_, the Fe^II^ and Fe^III^ ions are hexa- and tetra­coordinated, respectively. Each unique benzene-1,4-dicarbonitrile mol­ecule lies across a crystallographic inversion centre and bridges two Fe^II^ ions (each situated on an inversion centre), generating two-dimensional (4,4) square grid layers. The tetra­chloridoferrate(III) anions and nitro­methane solvent mol­ecules lie between the square grid layers and are further link to the adjacent layers into a three-dimensional supra­molecular structure through O—H⋯Cl and O—H⋯O hydrogen bonds.

## Related literature

For background to Fe^II^ spin-crossover complexes, see: Kahn & Martinez (1998 ▶); Neville *et al.* (2007 ▶, 2008 ▶); Murray (2008 ▶). For the use of two connecting organodinitrile ligands for the development of magnetism, see: Chainok *et al.* (2010 ▶, 2012 ▶). For the synthesis, see: Chainok *et al.* (2012 ▶).
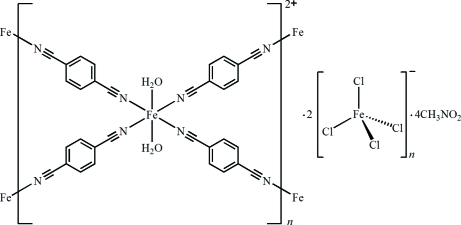


## Experimental

### 

#### Crystal data

[Fe(C_8_H_4_N_2_)_2_(H_2_O)_2_][FeCl_4_]_2_·4CH_3_NO_2_
*M* *_r_* = 987.62
Monoclinic, 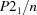

*a* = 12.1307 (9) Å
*b* = 12.1554 (9) Å
*c* = 13.8209 (10) Å
β = 102.466 (1)°
*V* = 1989.9 (3) Å^3^
*Z* = 2
Mo *K*α radiation
μ = 1.67 mm^−1^
*T* = 100 K
0.27 × 0.27 × 0.24 mm

#### Data collection

Bruker SMART APEX CCD area-detector diffractometer
Absorption correction: multi-scan (*SADABS*; Sheldrick, 1996 ▶) *T*_min_ = 0.661, *T*_max_ = 0.690
11573 measured reflections
4397 independent reflections
3541 reflections with *I* > 2σ(*I*)
*R*_int_ = 0.030

#### Refinement

*R*[*F* ^2^ > 2σ(*F* ^2^)] = 0.032
*wR*(*F* ^2^) = 0.073
*S* = 1.03
4397 reflections
233 parameters
3 restraints
H atoms treated by a mixture of independent and constrained refinement
Δρ_max_ = 0.76 e Å^−3^
Δρ_min_ = −0.50 e Å^−3^

### 

Data collection: *SMART* (Bruker, 2001 ▶); cell refinement: *SAINT* (Bruker, 2002 ▶); data reduction: *SAINT*; program(s) used to solve structure: *SHELXS97* (Sheldrick, 2008 ▶); program(s) used to refine structure: *SHELXL97* (Sheldrick, 2008 ▶); molecular graphics: *ORTEP-3 for Windows* (Farrugia, 1997 ▶) and *DIAMOND* (Brandenburg, 2006 ▶); software used to prepare material for publication: *publCIF* (Westrip, 2010 ▶).

## Supplementary Material

Crystal structure: contains datablock(s) global, I. DOI: 10.1107/S1600536812002486/tk5052sup1.cif

Supplementary material file. DOI: 10.1107/S1600536812002486/tk5052Isup2.cdx

Structure factors: contains datablock(s) I. DOI: 10.1107/S1600536812002486/tk5052Isup3.hkl

Additional supplementary materials:  crystallographic information; 3D view; checkCIF report

## Figures and Tables

**Table 1:** Selected bond lengths (Å)

Fe1—O1	2.0884 (15)
Fe1—N21	2.1309 (17)
Fe1—N11	2.1649 (17)
Cl1—Fe2	2.2070 (6)
Fe2—Cl2	2.1811 (6)
Fe2—Cl3	2.1968 (6)
Fe2—Cl4	2.1996 (6)

**Table 2:** Hydrogen-bond geometry (Å, °)

*D*—H⋯*A*	*D*—H	H⋯*A*	*D*⋯*A*	*D*—H⋯*A*
O1—H1⋯O2	0.87 (2)	1.99 (2)	2.804 (2)	155 (3)
O1—H1⋯O4	0.87 (2)	2.57 (3)	3.074 (3)	118 (2)
O1—H2⋯Cl1	0.84 (2)	2.43 (2)	3.2731 (16)	177 (3)

**Fig. 1.**
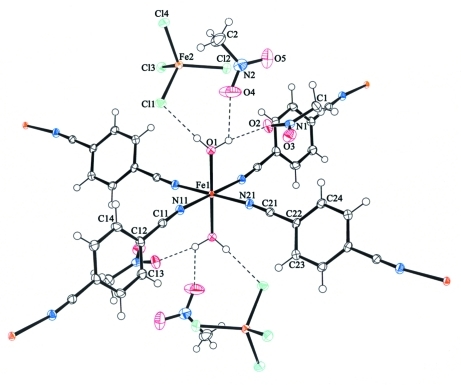
Displacement ellipsoid plot of a fragment at the 50% probability level containing the asymmetric unit with atom numbering and coordination environments of the metal centers of the title compound.

**Fig. 2.**
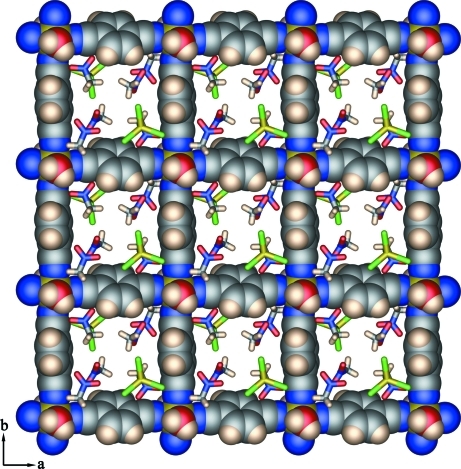
Inclusion of tetrachloridoferrate(III) anions and nitromethane molecules in the square grid networks of the title compound.

## supplementary crystallographic information

### Comment

The d^6^ Fe(II) complexes exhibiting spin-crossover (SCO) transitions between a ^1^A_1_ low spin (*S* = 0) and a ^5^T_2_ high spin (*S* = 2) states are of interest due to their possible applications as molecular switches or materials for information storage (Kahn & Martinez 1998). Although the fundamental origin of the SCO phenomenon is molecular, SCO transitions in the crystal structure can be reversibly switched by external stimuli such as light, temperature and pressure as well as the existence of long or short range supramolecular interactions (Neville *et al.* 2007, 2008; Murray 2008). The title compound was obtained as a minor product in another an earlier from part of further study of how the nature of the two-connecting organodinitrile bridging ligands affect the SCO phenomenon in the Fe(II) complexes (Chainok *et al.* 2010).

A fragment containing the asymmetric unit with atom numbering and coordination environments of the metal centre of the title compound is shown in Fig. 1. The asymmetric unit contains one iron(II) cation (half-occupancy), one coordinated water molecule, half of two independent benzene-1,4-dicarbonitrile ligand, one tetrachloridoferrate(III) anion, and two nitromethane solvent molecules. The Fe^II^ ion resides in an inversion center and is octahedrally coordinated by four nitrogen atoms from benzene-1,4-dicarbonitrile ligands in the equatorial plane and two equivalent terminal water molecules occupying the axial positions. The Fe—N and Fe—O bond lengths in the title compound, Table 1, are comparable to that observed for a high spin species in the corresponding Fe(II) complex containing the two-connecting organodinitrile ligands such as [Fe^II^(AIBN)(H_2_O)][Fe^III^Cl_4_]_2_ (Fe—N = 2.144 (1)–2.171 (1) Å and Fe–O = 2.084 (1) Å), AIBN = 2,2'-azobisisobutyronitrile (Chainok *et al.* 2010). Each benzene-1,4-dicarbonitrile ligand has crystallographically imposed inversion symmetry and is bound to two neighboring Fe^II^ ions generating two-dimensional approximate square grid layers with (4,4) topology perpendicular to the *c* axis, with the dimension of 12.1307 (9) × 12.1554 (9) Å (Fe^II^···Fe^II^ distances across the benzene-1,4-dicarbonitrile ligand), Fig. 2. The layers are packed to each other with an interlayer separation of 6.0777 (5) Å.

The Fe^III^ atom is a tetrahedrally coordinated to four chloride anions. The bond lengths and angles around the Fe(III) ions are within the common ranges for this type of coordination environment (Chainok *et al.* 2010). The tetrachloridoferrate(III) anions and the nitromethane solvent molecules all lie in general position in the structure, and are included in the layers by eschewing the interpenetration of the networks. These guest molecules are then further linked to the adjacent two-dimensional layers into a three-dimensional supramolecular structure through O—H···Cl and O—H···O hydrogen bonds formed between the apically water molecules coordinated to the metal ions and Cl and O atoms of the guest molecules, Table 2.

### Experimental

Single crystals of the title compound were obtained as the minor product during the synthesis of [Fe^II^(C_8_H_4_N_2_)_2_][Fe^III^Cl_4_]_2_ (Chainok *et al.* 2012) when traces of air and moisture are presented. Typically, FeCl_2_ (62 mg, 0.5 mmol) and FeCl_3_ (163 mg, 1 mmol) were dissolved in 3 ml of CH_3_NO_2_ to formed a yellow brown solution and this was pipetted into one side of the H-tube. Benzene-1,4-dicarbonitrile (192 mg, 1.5 mmol) was dissolved in 3 ml of CH_3_NO_2_ to give a colorless solution and this was pipetted into the other side arm of the H-tube. The H-tube was then carefully filled with CH_3_NO_2_. Upon slow diffusion for two weeks, yellow block-shaped of the major product (*ca*. 60% yield based on FeCl_2_) and pale yellow plate of the minor product (*ca*. 5% yield based on FeCl_2_) single crystals were formed in the iron-containing side of the H-tube.

### Refinement

The hydrogen atoms were placed in the geometrically idealized positions and constrained to ride on their parent atom positions with a C–H distances of 0.95 and 0.99 Å *U*_iso_(H) = 1.2*U*_eq_(C) and 0.98 Å for CH_3_ [*U*_iso_(H) = 1.5*U*_eq_(C)]. The hydrogen atoms attached to oxygen atoms of the water molecules were located in a difference Fourier map and refined being in their as-found positions with a *DFIX* restraint of O—H distance at 0.90 Å, with *U*_iso_(H) = 1.2*U*_eq_(O).

### Crystal data

**Table:**

[Fe(C_8_H_4_N_2_)_2_(H_2_O)_2_][FeCl_4_]_2_·4CH_3_NO_2_	*F*(000) = 988
*M**_r_* = 987.62	*D*_x_ = 1.648 Mg m^−^^3^
Monoclinic, *P*2_1_/*n*	Mo *K*α radiation, λ = 0.71073 Å
Hall symbol: -P 2yn	Cell parameters from 3583 reflections
*a* = 12.1307 (9) Å	θ = 2.4–27.4°
*b* = 12.1554 (9) Å	µ = 1.67 mm^−^^1^
*c* = 13.8209 (10) Å	*T* = 100 K
β = 102.466 (1)°	Block, yellow
*V* = 1989.9 (3) Å^3^	0.27 × 0.27 × 0.24 mm
*Z* = 2	

### Data collection

**Table:**

Bruker SMART APEX CCD area-detector diffractometer	4397 independent reflections
Radiation source: fine-focus sealed tube	3541 reflections with *I* > 2σ(*I*)
graphite	*R*_int_ = 0.030
Detector resolution: 8 pixels mm^-1^	θ_max_ = 28.1°, θ_min_ = 2.0°
ω and φ scans	*h* = −15→16
Absorption correction: multi-scan (*SADABS*; Sheldrick, 1996)	*k* = −12→15
*T*_min_ = 0.661, *T*_max_ = 0.690	*l* = −17→17
11573 measured reflections	

### Refinement

**Table:**

Refinement on *F*^2^	Primary atom site location: structure-invariant direct methods
Least-squares matrix: full	Secondary atom site location: difference Fourier map
*R*[*F*^2^ > 2σ(*F*^2^)] = 0.032	Hydrogen site location: inferred from neighbouring sites
*wR*(*F*^2^) = 0.073	H atoms treated by a mixture of independent and constrained refinement
*S* = 1.03	*w* = 1/[σ^2^(*F*_o_^2^) + (0.042*P*)^2^] where *P* = (*F*_o_^2^ + 2*F*_c_^2^)/3
4397 reflections	(Δ/σ)_max_ = 0.001
233 parameters	Δρ_max_ = 0.76 e Å^−^^3^
3 restraints	Δρ_min_ = −0.50 e Å^−^^3^

### Special details

**Table:**

Geometry. All e.s.d.'s (except the e.s.d. in the dihedral angle between two l.s. planes) are estimated using the full covariance matrix. The cell e.s.d.'s are taken into account individually in the estimation of e.s.d.'s in distances, angles and torsion angles; correlations between e.s.d.'s in cell parameters are only used when they are defined by crystal symmetry. An approximate (isotropic) treatment of cell e.s.d.'s is used for estimating e.s.d.'s involving l.s. planes.
Refinement. Refinement of *F*^2^ against all reflections. The weighted *R*-factor *wR* and goodness of fit *S* are based on *F*^2^, conventional *R*-factors *R* are based on *F*, with *F* set to zero for negative *F*^2^. The threshold expression of *F*^2^ > σ(*F*^2^) is used only for calculating *R*-factors(gt) *etc*. and is not relevant to the choice of reflections for refinement. *R*-factors based on *F*^2^ are statistically about twice as large as those based on *F*, and *R*- factors based on ALL data will be even larger.

### Fractional atomic coordinates and isotropic or equivalent isotropic displacement parameters (Å^2^)

**Table:**

	*x*	*y*	*z*	*U*_iso_*/*U*_eq_	
Fe1	0.5000	0.5000	0.5000	0.01056 (10)	
Cl1	0.31456 (5)	0.45491 (4)	0.76369 (4)	0.02217 (13)	
O1	0.52584 (13)	0.46637 (12)	0.65141 (11)	0.0173 (3)	
N1	0.74782 (15)	0.26095 (15)	0.71549 (13)	0.0200 (4)	
C1	0.7964 (2)	0.15057 (19)	0.74355 (17)	0.0270 (5)	
H1A	0.7828	0.1301	0.8086	0.040*	
H1B	0.8778	0.1522	0.7465	0.040*	
H1C	0.7606	0.0965	0.6941	0.040*	
H1	0.576 (2)	0.417 (2)	0.676 (2)	0.052 (9)*	
Fe2	0.29049 (3)	0.29511 (2)	0.83208 (2)	0.01702 (9)	
Cl2	0.42489 (5)	0.18565 (5)	0.80885 (4)	0.02811 (14)	
O2	0.64553 (13)	0.26786 (12)	0.68614 (12)	0.0259 (4)	
N2	0.67018 (18)	0.34984 (17)	0.93557 (15)	0.0297 (5)	
C2	0.6206 (3)	0.3774 (2)	1.0194 (2)	0.0437 (7)	
H2A	0.6721	0.3550	1.0810	0.066*	
H2B	0.5485	0.3388	1.0131	0.066*	
H2C	0.6080	0.4570	1.0205	0.066*	
H2	0.4716 (18)	0.466 (2)	0.6803 (19)	0.043 (9)*	
Cl3	0.12062 (5)	0.23479 (4)	0.76444 (4)	0.02225 (13)	
O3	0.81115 (14)	0.33973 (14)	0.72375 (13)	0.0318 (4)	
Cl4	0.30172 (5)	0.31921 (5)	0.99164 (4)	0.02609 (14)	
O4	0.6489 (2)	0.41206 (17)	0.86466 (15)	0.0661 (7)	
O5	0.72545 (17)	0.26586 (17)	0.93885 (17)	0.0515 (6)	
N11	0.49078 (14)	0.32436 (14)	0.47352 (13)	0.0159 (4)	
C11	0.49325 (17)	0.23044 (17)	0.47862 (15)	0.0151 (4)	
C12	0.49595 (17)	0.11201 (16)	0.48873 (15)	0.0164 (4)	
C13	0.48332 (19)	0.06644 (17)	0.57797 (16)	0.0203 (5)	
H13	0.4724	0.1125	0.6306	0.024*	
C14	0.51318 (19)	0.04656 (17)	0.41060 (16)	0.0200 (5)	
H14	0.5224	0.0791	0.3503	0.024*	
N21	0.32197 (14)	0.50641 (13)	0.48775 (13)	0.0166 (4)	
C21	0.22841 (17)	0.50554 (16)	0.48975 (15)	0.0148 (4)	
C22	0.11031 (17)	0.50257 (16)	0.49328 (15)	0.0146 (4)	
C23	0.07732 (17)	0.42932 (16)	0.55939 (15)	0.0160 (4)	
H23	0.1310	0.3821	0.5994	0.019*	
C24	0.03464 (17)	0.57343 (17)	0.43427 (15)	0.0164 (4)	
H24	0.0595	0.6230	0.3903	0.020*	

### Atomic displacement parameters (Å^2^)

**Table:**

	*U*^11^	*U*^22^	*U*^33^	*U*^12^	*U*^13^	*U*^23^
Fe1	0.0092 (2)	0.00766 (19)	0.0156 (2)	0.00009 (14)	0.00442 (15)	0.00041 (15)
Cl1	0.0274 (3)	0.0176 (3)	0.0228 (3)	−0.0022 (2)	0.0083 (2)	0.0030 (2)
O1	0.0169 (8)	0.0178 (8)	0.0186 (8)	0.0021 (6)	0.0067 (6)	0.0021 (6)
N1	0.0243 (11)	0.0234 (10)	0.0130 (9)	0.0027 (8)	0.0053 (8)	0.0015 (7)
C1	0.0316 (14)	0.0252 (13)	0.0236 (12)	0.0121 (10)	0.0046 (10)	0.0056 (10)
Fe2	0.02082 (17)	0.01520 (16)	0.01655 (17)	0.00175 (12)	0.00737 (13)	0.00109 (12)
Cl2	0.0319 (3)	0.0281 (3)	0.0275 (3)	0.0120 (2)	0.0134 (3)	0.0028 (2)
O2	0.0192 (8)	0.0229 (8)	0.0328 (9)	0.0043 (6)	−0.0009 (7)	−0.0019 (7)
N2	0.0383 (13)	0.0251 (11)	0.0270 (11)	−0.0068 (9)	0.0104 (9)	−0.0065 (9)
C2	0.072 (2)	0.0264 (14)	0.0435 (17)	0.0025 (13)	0.0369 (16)	−0.0013 (12)
Cl3	0.0246 (3)	0.0183 (3)	0.0254 (3)	−0.0026 (2)	0.0088 (2)	−0.0022 (2)
O3	0.0274 (10)	0.0317 (10)	0.0394 (10)	−0.0080 (7)	0.0139 (8)	−0.0008 (8)
Cl4	0.0343 (3)	0.0283 (3)	0.0175 (3)	0.0041 (2)	0.0098 (2)	0.0008 (2)
O4	0.132 (2)	0.0412 (13)	0.0303 (11)	−0.0064 (13)	0.0280 (13)	0.0054 (10)
O5	0.0399 (12)	0.0445 (12)	0.0723 (15)	0.0128 (9)	0.0172 (11)	−0.0088 (11)
N11	0.0160 (9)	0.0117 (9)	0.0203 (9)	−0.0003 (6)	0.0046 (7)	−0.0003 (7)
C11	0.0140 (10)	0.0146 (11)	0.0176 (10)	−0.0011 (8)	0.0055 (8)	−0.0002 (8)
C12	0.0167 (10)	0.0095 (9)	0.0228 (11)	−0.0021 (8)	0.0041 (9)	−0.0010 (8)
C13	0.0283 (12)	0.0125 (10)	0.0219 (11)	−0.0007 (8)	0.0097 (9)	−0.0039 (9)
C14	0.0285 (12)	0.0131 (10)	0.0197 (11)	−0.0022 (9)	0.0081 (9)	0.0017 (9)
N21	0.0145 (9)	0.0137 (9)	0.0223 (9)	−0.0001 (7)	0.0058 (7)	−0.0005 (7)
C21	0.0165 (11)	0.0118 (10)	0.0169 (10)	0.0000 (8)	0.0053 (8)	−0.0001 (8)
C22	0.0109 (9)	0.0154 (10)	0.0186 (10)	−0.0018 (7)	0.0054 (8)	−0.0041 (8)
C23	0.0147 (10)	0.0149 (10)	0.0178 (10)	0.0018 (8)	0.0018 (8)	−0.0010 (8)
C24	0.0157 (10)	0.0151 (10)	0.0198 (11)	−0.0022 (8)	0.0071 (9)	−0.0003 (8)

### Geometric parameters (Å, °)

**Table:**

Fe1—O1	2.0884 (15)	N2—C2	1.455 (3)
Fe1—O1^i^	2.0884 (15)	C2—H2A	0.9800
Fe1—N21^i^	2.1309 (17)	C2—H2B	0.9800
Fe1—N21	2.1309 (17)	C2—H2C	0.9800
Fe1—N11	2.1649 (17)	N11—C11	1.144 (3)
Fe1—N11^i^	2.1649 (17)	C11—C12	1.446 (3)
Cl1—Fe2	2.2070 (6)	C12—C13	1.390 (3)
O1—H1	0.872 (17)	C12—C14	1.393 (3)
O1—H2	0.840 (16)	C13—C14^ii^	1.382 (3)
N1—O3	1.218 (2)	C13—H13	0.9500
N1—O2	1.222 (2)	C14—C13^ii^	1.382 (3)
N1—C1	1.483 (3)	C14—H14	0.9500
C1—H1A	0.9800	N21—C21	1.141 (3)
C1—H1B	0.9800	C21—C22	1.444 (3)
C1—H1C	0.9800	C22—C24	1.388 (3)
Fe2—Cl2	2.1811 (6)	C22—C23	1.395 (3)
Fe2—Cl3	2.1968 (6)	C23—C24^iii^	1.380 (3)
Fe2—Cl4	2.1996 (6)	C23—H23	0.9500
N2—O5	1.217 (3)	C24—C23^iii^	1.380 (3)
N2—O4	1.221 (3)	C24—H24	0.9500
			
O1—Fe1—O1^i^	180.0	Cl4—Fe2—Cl1	109.08 (2)
O1—Fe1—N21^i^	89.02 (6)	O5—N2—O4	124.7 (2)
O1^i^—Fe1—N21^i^	90.98 (6)	O5—N2—C2	118.9 (2)
O1—Fe1—N21	90.98 (6)	O4—N2—C2	116.4 (2)
O1^i^—Fe1—N21	89.02 (6)	N2—C2—H2A	109.5
N21^i^—Fe1—N21	180.00 (9)	N2—C2—H2B	109.5
O1—Fe1—N11	88.13 (6)	H2A—C2—H2B	109.5
O1^i^—Fe1—N11	91.87 (6)	N2—C2—H2C	109.5
N21^i^—Fe1—N11	89.53 (6)	H2A—C2—H2C	109.5
N21—Fe1—N11	90.47 (6)	H2B—C2—H2C	109.5
O1—Fe1—N11^i^	91.87 (6)	C11—N11—Fe1	166.95 (17)
O1^i^—Fe1—N11^i^	88.13 (6)	N11—C11—C12	177.9 (2)
N21^i^—Fe1—N11^i^	90.47 (6)	C13—C12—C14	121.59 (19)
N21—Fe1—N11^i^	89.53 (6)	C13—C12—C11	118.55 (19)
N11—Fe1—N11^i^	180.0	C14—C12—C11	119.85 (19)
Fe1—O1—H1	118.0 (19)	C14^ii^—C13—C12	119.4 (2)
Fe1—O1—H2	120.9 (19)	C14^ii^—C13—H13	120.3
H1—O1—H2	111 (2)	C12—C13—H13	120.3
O3—N1—O2	123.50 (19)	C13^ii^—C14—C12	119.0 (2)
O3—N1—C1	118.69 (19)	C13^ii^—C14—H14	120.5
O2—N1—C1	117.81 (18)	C12—C14—H14	120.5
N1—C1—H1A	109.5	C21—N21—Fe1	173.63 (17)
N1—C1—H1B	109.5	N21—C21—C22	179.0 (2)
H1A—C1—H1B	109.5	C24—C22—C23	122.29 (19)
N1—C1—H1C	109.5	C24—C22—C21	119.98 (19)
H1A—C1—H1C	109.5	C23—C22—C21	117.71 (18)
H1B—C1—H1C	109.5	C24^iii^—C23—C22	118.85 (19)
Cl2—Fe2—Cl3	113.33 (3)	C24^iii^—C23—H23	120.6
Cl2—Fe2—Cl4	110.02 (2)	C22—C23—H23	120.6
Cl3—Fe2—Cl4	108.69 (2)	C23^iii^—C24—C22	118.86 (19)
Cl2—Fe2—Cl1	107.56 (2)	C23^iii^—C24—H24	120.6
Cl3—Fe2—Cl1	108.07 (2)	C22—C24—H24	120.6
			
O1—Fe1—N11—C11	−10.8 (7)	O1—Fe1—N21—C21	−13.1 (15)
O1^i^—Fe1—N11—C11	169.2 (7)	O1^i^—Fe1—N21—C21	166.9 (15)
N21^i^—Fe1—N11—C11	78.2 (7)	N21^i^—Fe1—N21—C21	141 (2)
N21—Fe1—N11—C11	−101.8 (7)	N11—Fe1—N21—C21	75.1 (15)
N11^i^—Fe1—N11—C11	−43 (18)	N11^i^—Fe1—N21—C21	−104.9 (15)
Fe1—N11—C11—C12	25 (7)	Fe1—N21—C21—C22	−35 (15)
N11—C11—C12—C13	2(6)	N21—C21—C22—C24	−168 (13)
N11—C11—C12—C14	−177 (100)	N21—C21—C22—C23	14 (14)
C14—C12—C13—C14^ii^	−0.6 (4)	C24—C22—C23—C24^iii^	0.6 (3)
C11—C12—C13—C14^ii^	−179.35 (19)	C21—C22—C23—C24^iii^	178.61 (18)
C13—C12—C14—C13^ii^	0.6 (4)	C23—C22—C24—C23^iii^	−0.6 (3)
C11—C12—C14—C13^ii^	179.33 (19)	C21—C22—C24—C23^iii^	−178.57 (18)

Symmetry codes: (i) −*x*+1, −*y*+1, −*z*+1; (ii) −*x*+1, −*y*, −*z*+1; (iii) −*x*, −*y*+1, −*z*+1.

### Hydrogen-bond geometry (Å, °)

**Table:**

*D*—H···*A*	*D*—H	H···*A*	*D*···*A*	*D*—H···*A*
O1—H1···O2	0.87 (2)	1.99 (2)	2.804 (2)	155 (3)
O1—H1···O4	0.87 (2)	2.57 (3)	3.074 (3)	118 (2)
O1—H2···Cl1	0.84 (2)	2.43 (2)	3.2731 (16)	177 (3)

## References

[bb1] Brandenburg, K. (2006). *DIAMOND* Crystal Impact GbR, Bonn, Germany.

[bb2] Bruker (2001). *SMART* Bruker AXS Inc., Madison, Wisconsin, USA.

[bb3] Bruker (2002). *SAINT* Bruker AXS Inc., Madison, Wisconsin, USA.

[bb4] Chainok, K., Harding, D. J., Moubaraki, B., Batten, S. R. & Murray, S. R. (2012). Unpublished results.

[bb5] Chainok, K., Neville, S. M., Moubaraki, B., Batten, S. R., Murray, K. S., Forsyth, C. M., Cashion, J. D. & Haller, K. J. (2010). *Dalton Trans.* **39**, 10900–10909. 10.1039/c0dt00447b 20721391

[bb6] Farrugia, L. J. (1997). *J. Appl. Cryst.* **30**, 565.

[bb7] Kahn, O. & Martinez, C. J. (1998). *Science*, **279**, 44–48.

[bb8] Murray, K. S. (2008). *Eur. J. Inorg. Chem.* pp. 3101–3121.

[bb9] Neville, S. M., Leita, B. A., Halder, G. J., Kepert, C. J., Moubaraki, B., Létard, J.-F. & Murray, K. S. (2008). *Chem. Eur. J.* **14**, 10123–10133. 10.1002/chem.200800886 18803203

[bb10] Neville, S. M., Leita, B. A., Offermann, D. A., Duriska, M. B., Moubaraki, B., Chapman, K. W., Halder, J. D. & Murray, K. S. (2007). *Eur. J. Inorg. Chem.* pp. 1073–1085.

[bb11] Sheldrick, G. M. (1996). *SADABS* University of Göttingen, Germany.

[bb12] Sheldrick, G. M. (2008). *Acta Cryst.* A**64**, 112–122. 10.1107/S0108767307043930 18156677

[bb13] Westrip, S. P. (2010). *J. Appl. Cryst.* **43**, 920–925.

